# The single-cell sequencing: new developments and medical applications

**DOI:** 10.1186/s13578-019-0314-y

**Published:** 2019-06-26

**Authors:** Xiaoning Tang, Yongmei Huang, Jinli Lei, Hui Luo, Xiao Zhu

**Affiliations:** 1 The Marine Biomedical Research Institute, Guangdong Medical University, Zhanjiang, 524023 China; 2 Guangdong Provincial Zhanjiang Bay Key Laboratory, Zhanjiang, 524023 China

**Keywords:** Cancer, Clinical applications, Developments, Immunology, Single-cell sequencing technologies, Transcriptome

## Abstract

Single-cell sequencing technologies can be used to detect the genome, transcriptome and other multi-omics of single cells. They can show the differences and evolutionary relationships of various cells. This review introduces the latest advances in single-cell sequencing technologies and their applications in oncology, microbiology, neurology, reproduction, immunology, digestive and urinary systems, highlighting the important role that single-cell sequencing techniques play in these areas.

## Background

Single-cell sequencing technologies refer to the sequencing of a single-cell genome or transcriptome, so as to obtain genomic, transcriptome or other multi-omics information to reveal cell population differences and cellular evolutionary relationships. Traditional sequencing methods can only get the average of many cells, unable to analyze a small number of cells and lose cellular heterogeneity information. Compared with traditional sequencing technology, single-cell technologies have the advantages of detecting heterogeneity among individual cells [1], distinguishing a small number of cells, and delineating cell maps. In 2013, it was named “Nature Methods” as the annual technology [2]. However, early single-cell sequencing limited its widespread use due to its high cost. But as the research progressed, many new single-cell sequencing methods were developed that reduced the cost threshold for single-cell sequencing. Nowadays, single-cell sequencing technology is increasingly used in various fields. This review describes recent advances in single-cell sequencing methods and their applications in tumors, microbiology, neurology, reproduction, immunity, digestion, and urinary systems, and clarifies the important role of single-cell sequencing technologies in basic and clinical research.

## Single-cell sequencing methods and recent developments

### Development of single-cell sequencing methods

**Fig. 1:**
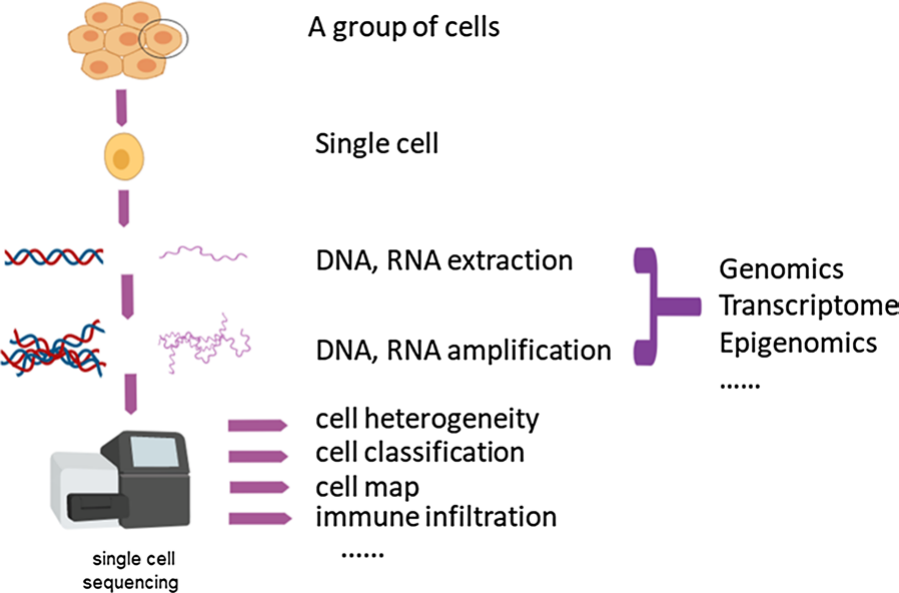
The principle of single-cell sequencing. It is a process of isolating a single cell for sequencing and studying cell heterogeneity, molecular mapping, immune infiltration and epigenetic changes

As research continues to deepen, the capabilities of single-cell sequencing methods (Fig. 1) continue to increase and evolve toward lower detection costs, advancing scientists research on the molecular mechanisms at the single-cell level. Vitak et al. [3] proposed a single-cell combinatorial marker sequencing technique (SCI-seq) that can simultaneously construct thousands of single-cell libraries and detect variations in somatic cell copy number (Table 1). This technique increases the number of cells detected and reduces the cost of library construction, and has important value in the study of somatic cell variation. Chen et al. [4] developed a novel single-cell whole-genome amplification method that can detect CNV at kilobase resolution and more effectively detect mutations in more diseases (Table 1). Guo et al. [5] developed a single-cell multiple sequencing technique (scCOOL-seq) that allows simultaneous analysis of single-cell chromatin state/nuclear niche localization, copy number variations, ploidy and DNA methylation, which can indicate different functions and patterns of chromatin state and DNA methylation (Table 1). Casasent et al. [6] invented a Topographic Single Cell Sequencing (TSCS) that provides accurate spatial location information for cells (Table 1). This technique accurately measures and describes the specific characteristics of individual tumor cells spatially and helps to study the invasion and metastasis of tumor cells. Demaree et al. [7] describe a high-throughput and low-deviation single-cell sequencing (SiC-seq) method that uses droplet microfluidics to separate, amplify, and barcode the genome of a single cell (Table 1). This approach enables broader genomic studies for different cell populations. The Microwell-seq developed by Han et al. is a high-throughput and low-cost scRNA-seq platform [8] (Table 1). Not only does it improve the detection abundance of single-cell technologies, but it also reduces the cost of detection by an order of magnitude compared to single-cell sequencing techniques coated with oil droplets. The SPLit-seq technology from Rosenberg et al., based on the principle of a low-cost combined barcode, can reduce the cost of single-cell transcriptome sequencing to 1 cent. Once again broke the cost threshold for single cell detection [9] (Table 1).

**Table 1:** Single-cell sequencing technologies

Single-cell sequencing	Characteristics	Functions
Separate application		
SCI-seq^3^	Single-cell combination marker	Construction of single-cell libraries and detection of cell copy number variation
LIANTI^4^	Single cell whole genome amplification	Detection of cell copy number variation and disease-related mutations
scCOOL-seq^5^	Single cell multiplex sequencing	Detection of chromatin status/nucleosome localization, DNA methylation, copy number variation and ploidy
TSCS^6^	Provide accurate spatial location information	Describe the spatial characteristics of individual tumor cells
SiC-seq^7^	High throughput and low deviation	Extensive genomic research on different cells
Microwell-seq^8^	High throughput and low cost	Improve the detection abundance of single cell sequencing technology
SPLit-seq^9^	Combine barcode principle and low cost	Single cell transcriptome sequencing
Joint application		
CROP-seq^10^	High throughput	Analysis of complex regulatory mechanisms and functions of heterogeneous cell populations
CRISPRi + scRNA-seq^11^	High throughput	Analyze the function of regulatory elements and the relationship between regulatory elements and cells
Single-Nucleus RNA-Seq +DroNc-Seq^12^	High sensitivity and high cell sorting efficiency	A variety of cells can be accurately analyzed. It may be used in the Human Cell Atlas Project in the future
snDrop-seq + scTHS-seq^13^	High throughput	It can be used to detect nuclear transcripts and epigenetic features, or related analysis of frozen tissue in humans

### The joint use of single-cell sequencing technologies

The single-cell sequencing detection cost reduction is beneficial to the combination of other technologies and single-cell sequencing technologies, greatly improving the efficiency of single-cell detection. Datlinger et al. [10] combined CRISPR screening with single-cell RNA sequencing to invent CROP-seq (Table 1), which enables high-throughput functional analysis of complex regulatory mechanisms and heterogeneous cell populations. Gasperini et al. [11] combines CRISPRi and scRNA-seq to facilitate the study of the function of regulatory elements and the interrelationship between regulatory elements and genes (Table 1). Habib et al. [12] combined sNuc-Seq with microfluidic technology to introduce a single-cell nuclear RNA sequencing method with low cost, high-sensitive and high-efficiency cell classification, which is expected to be used in human cell mapping projects (Table 1). Lake et al. [13] combined single-core sequencing (snDrop-seq) and single-cell transposon hypersensitive site sequencing (scTHS-seq) to create a high-throughput sequencing platform for parallel detection of nuclear transcripts and epigenetic features (Table 1). It provides a way to comprehensively analyze gene expression and regulation in cryopreserved samples of human tissues.

The above studies (Table 1) show that single-cell sequencing technologies were constantly updated and developed, providing a technical basis for the construction of a complete cell map, greatly promoting the single-cell research process. In the future, single-cell sequencing technologies integrated multi-omics methods may become popular, playing an increasingly powerful role in single-cell research of complex organs and tissues, diagnosis and treatment of clinical diseases.

## Single-cell sequencing applications

### Applications in cancer

**Fig. 2:**
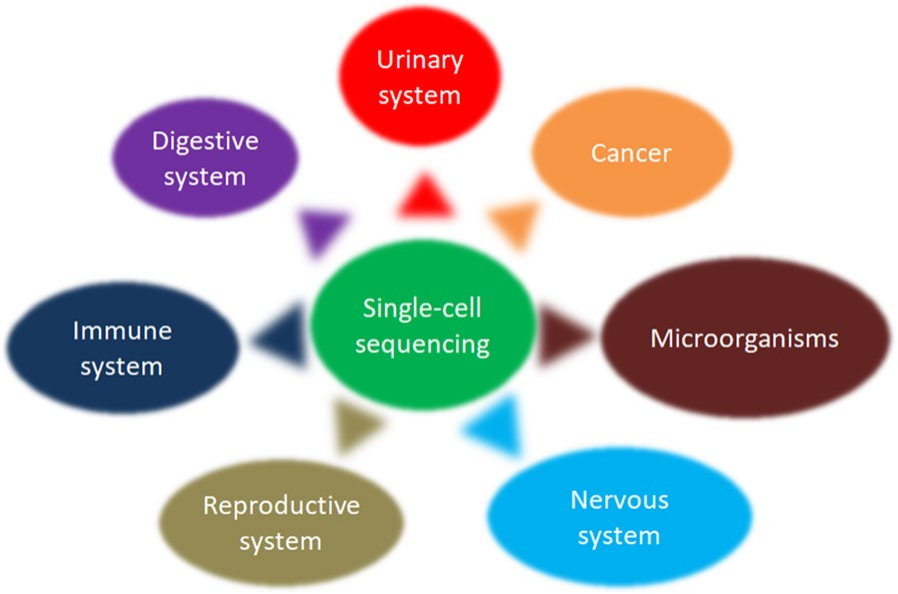
The clinical applications of single-cell sequencing in different fields, like cancer, immune system, reproductive system, microorganisms, etc. These will help to clarify some of the problems and provide a basis for finding better treatments

Studies have shown that genetic or genomic variation can lead to cells with different genetic and phenotypic characteristics within the tumor tissue, making the tumor tissue highly heterogeneous [14, 15]. This high degree of heterogeneity may be related to the mechanisms of tumorigenesis [16] and metastasis [17–19], so researchers need to perform more accurate analysis of tumor cells. Traditional sequencing methods can only detect cell populations, get the average of the signals in a group of cells, but the heterogeneity in tumor cells can be masked. Therefore, traditional sequencing methods do not study tumor cells well. Single-cell sequencing technologies can perfectly compensate for the shortcomings of traditional sequencing methods. The cell map of tumor cells and tumor microenvironment was drawn by detecting the heterogeneity of tumor cells. Further, clarify the cell group within the tumor tissue and find specific markers, as well as explain a series of problems such as tumorigenesis and metastasis. Therefore, single-cell sequencing technologies were widely used in the research of various tumors, and were of great significance for the development of new diagnostic and anti-tumor treatment methods (Fig. 2).

### Colorectal cancer

Zhang et al. [20] mapped the T cell immunoreceptor of colorectal cancer by single-cell sequencing technology, and revealed the subgroup classification, tissue distribution characteristics, tumor heterogeneity and drug target gene expression of colorectal cancer T cells. A potential state transition relationship between T cell populations and subpopulations distributed across tissues was identified. In the same year, Bian et al. [21] used this technique to analyze the characteristics and relationship of genomic copy number variation, DNA methylation abnormality and gene expression change during the occurrence and metastasis of human colorectal cancer from single cell resolution and multi-group level. The above studies are of great significance for revealing the new mechanism of human colon cancer and improving the diagnosis and treatment of colorectal cancer.

### Breast cancer

#### The origin and transformation of breast cancer

Breast cancer is a highly heterogeneous disease caused by genetically altered mammary epithelial cells. Nguyen et al. [22–24] mapped the single cell pattern of human mammary epithelial cells by single-cell sequencing technology and analyzed the diversity and status of cell types in breast cells. The findings help to understand the early origins of breast cancer and provide the basis for improving the early detection of cancer and preventing cancer progression. Casasent et al. [6] reported the polyclonal origin of breast cancer and the mechanism of transformation between ductal carcinoma in situ and invasive carcinoma by topographic single-cell sequencing. Direct genomic lineages between in situ tumor subpopulations and invasive tumor subpopulations are revealed in this study. Furthermore, the time and reason for the occurrence of subcloning and the mechanisms of the formation of invasive carcinoma due to the intra-ductal cloning into adjacent tissues were further demonstrated. The results of this study provide a theoretical basis for preventing early breast cancer from turning into invasive cancer.

#### Breast cancer microenvironment

The single-cell technologies explored the immune microenvironment of breast cancer and finds immunizing changes. Azizi et al. [25] mapped a single-cell map of various immune cells in the microenvironment of breast tumor. Through this map, the gene expression characteristics of human breast tumors and paired normal tissues, lymph node tissues and peripheral blood were analyzed. It was found that the heterogeneity of intratumoral lymphocytes and myeloid cells can induce combined gene expression through environmental stimulation. Based on T cell receptor (TCR) and T cell matched single-cell RNA sequencing data, the T cell continuous activation profile was inconsistent with the polarization model of macrophage in cancer. This study examined the microenvironment of breast cancer and helped to differentiate its immune cells and to identify the differences in gene expression of tumor-infiltrating immune cells and the causes of differences.

#### Breast cancer and estrogen

The researchers observed a close relationship between estrogen and breast cancer. Estrogen activates estrogen receptor alpha (ERα) to drive the progression of breast cancer. Zhu et al. [26] studied the estrogen signaling pathway and the mechanism of ERα-regulated transcription by single-cell transcriptome sequencing, and found the dynamic transcriptional heterogeneity of ERα-positive breast cancer cells after estrogen stimulation. This study reveals the metabolic pathways involved in estrogen-mediated monocarbon, purine and polyamine synthesis in breast cancer, and the discovery that PPAT and AZIN1 were direct ERa targets for the cell survival and growth of breast cancer. It supplements the pathogenesis of breast cancer and provides a theoretical basis for the study of tumor-targeted drugs.

#### Brain tumor

It is generally believed that brain astrocytomas and oligodendrogliomas originate from different types of cells. However, Venteicher et al. [27] found that IDH mutant gliomas share a common origin through single-cell sequencing technology. The difference between them is mainly the difference in genetics and the composition of the tumor microenvironment. Two of the different subtypes of tumors have universally similar stem cell programs, which can be distinguished from similar neuroglial mass spectrometry systems. Tirosh et al. [28] identified cancer stem cells and their differentiated progeny through a single cell map in human oligodendroglioma. The findings support the cancer stem cell hypothesis and confirm that cancer stem cells were the main source of growth for oligodendroglioma. These studies suggest that these cells may become new therapeutic targets. Researcher Suvà suggested that immunotherapy can be used to attack specific cell types, thereby terminating tumor growth [27]. It is of great significance for the treatment of such diseases.

#### Head and neck cell carcinoma

Puram et al. [29] analyzed the ecosystem of head and neck squamous cell carcinoma (HNSCC) by single-cell sequencing technology, delineating the first cell map of head and neck tumors and revealing the types of head and neck tumors and their metastasis-related procedures. This study provides a new understanding of the causes and metastasis of tumor metastasis, and helps to find new targets for blocking tumor metastasis.

#### Hematological tumors

Single-cell sequencing technologies help to find classification markers and analyze the causes of disease occurrence in hematological tumors with high heterogeneity, providing a theoretical basis for clinical diagnosis and treatment of diseases.

Ledergor et al. [30] found at the single-cell level that there were common expressions and differences in certain genes among different myeloma plasma cell samples. New markers can be sought by these common expressions. Differences in gene expression patterns and chromosome structure among the samples may be responsible for the high heterogeneity of myeloma plasma cells. This study summarizes the positive role of the single-cell approach in the diagnosis, treatment, and prognosis of myeloma. Peripheral blood is used to distinguish tumor cells and diagnose diseases. It can not only facilitate the collection of samples but also reduce the pain of patients during the collection of samples.

In the study of acute leukemia, Ley et al. [16] found genes associated with AML, and measured 9 mutations in each tumor sample cell. The results suggest that these heterogeneities caused by genetic mutations may be related to pathogenesis. Hou et al. [31] performed a whole exome single-cell sequencing on cells from patients with JAK2-negative myeloproliferative tumors, and the results indicated that this tumor represents a monoclonal evolution. They further identified candidate mutations associated with essential thrombocythemia (ET) and speculated that these mutations may be involved in tumor progression. De Bie et al. [32] demonstrated the sequence of mutations in T cell acute lymphoblastic leukemia (T-ALL) by targeted single-cell sequencing, and T-ALL development can begin in pluripotent progenitor cells.

#### Liver cancer and lung cancer

Chinese researchers at the single-cell level mapped the immunological map of the liver cancer microenvironment and the T cell immune map of lung cancer [33, 34]. The subgroup classification, tissue distribution characteristics, tumor heterogeneity and drug target gene expression of immune cells in liver cancer and lung cancer were revealed. The above studies are helpful to understand the immune microenvironment of liver cancer and lung cancer, to find effective biomarkers, new tumor immunotherapy, and drug targets. It is of great significance for the diagnosis and treatment of liver cancer and lung cancer.

Despite a large number of microorganisms in the world, researchers have only studied a small fraction of the microbial species. Due to the extremely small sample size of most microorganisms and the difficulty of culture, it is very difficult to study the microbial genome structure. However, the emergence of single-cell sequencing has led to important breakthroughs in the microbiology field. Single-cell sequencing technology provides cell-specific genetic information that is lacking in traditional genome-wide studies of bacteria. It also discovers genes associated with bacterial life activities and classifies microorganisms by genomic models [35]. Lan et al. [36] performed high-throughput single-cell genome sequencing (SiC-seq) on bacterial and fungal synthetic communities to analyze the distribution of antibiotic resistance genes, virulence factors and phage sequences in environmental microbial communities. This study enabled the microbial single-cell sequencing of prokaryotes. In the future, this technology may isolate low titer pathogenic bacterial samples and sequence them at the single-cell level to examine their virulence genotype sources [7]. According to the above studies, single-cell sequencing technologies play important roles in the classification, evolution and drug resistance of microorganisms. It is good for mining meaningful environmental microbes and finding ways to resist antimicrobial resistance.

## Applications in the nervous system

### Nerve cell typing

In the nervous system, there is a difference among individual neurons because there are some unique copy number variations in nerve cells [37]. The heterogeneity among these neurons makes it difficult to study how brain circuits are formed and to resolve neuronal reconnections. However, single-cell sequencing technology can study many different periods of nerve cells, and draw a detailed single-cell map to understand and identify different types of neurons and their connecting molecules in the brain [38]. Luo et al. [39] differentiated subtypes of mouse and human frontal cortical neuronal cells by high-throughput single-cell methylation sequencing. They identified a new set of neurons in the human frontal cortex and redefine the neuron type based on the methylation group of neurons. Lake et al. [40] used a new single-cell nuclear sequencing method to map the second generation single cell of an adult brain. A variety of different neurons, glial cell subtypes, and cell subtypes that are more susceptible to common risk factors for different brain diseases are found. It is expected to understand the pathogenesis of neurological diseases.

### Applications of brain nerve development and regeneration process

Single-cell sequencing technologies can study the types of brain cells and the relationships among cells during development (Fig. 2). Fan et al. [41] identified multiple subpopulations of cells in each region in multiple regions of human metaphase embryos by single-cell sequencing techniques, and analyzed gene expression and neuronal maturation in these regions. Zhong et al. [42] mapped a single-cell transcriptome map of human prefrontal embryonic development through single-cell sequencing. From this map, the diversity of cell types in the prefrontal lobe of the human embryonic brain and the developmental relationship among different cell types were analyzed, and the molecular regulation mechanism of neuron production and loop formation was further revealed. It is important to study the function of key cell types and to map intact human brain cells. Carter et al. [43] identified a major subset of cerebellar cells and a subpopulation that is beneficial to cerebellar development in a mouse cerebellar development map drawn by single-cell sequencing. These studies will facilitate future research on cerebellar development, neurobiology and diseases.

## Applications in the field of reproductive and embryonic medicine

The single-cell sequencing technologies can sequence and quantify the whole genome of germ cells and embryonic cells at the single-cell level (Fig. 2). This will help to understand the occurrence of germ cells and the screening, diagnosis, and treatment of reproductive and genetic diseases.

### Germ cell

Chen et al. [44] revealed the dynamic process and molecular characteristics of gene expression during spermatogenesis in mice and the specific patterns of alternative splicing through single-cell sequencing technology, as well as the discovery of key regulators for specific stages of male germ cell development. In the same year, the team performed single-cell RNA sequencing (scRNA-seq) analysis of human normal testicular cells and abnormal testicular cells. Based on the results, a hierarchical model of spermatogonial subtypes, spermatocyte subtypes and sperm cell subtypes was established, and specific markers for human germ cells were further discovered. In addition, changes of expression patterns in testicular somatic cells in an NOA patient were identified, which may be the pathogenesis of NOA [45]. The above studies provide valuable data sources for the development and maturation of mammalian sperm and the development of gametes. It also contributes to the understanding of male infertility and the treatment of such diseases.

### Reproductive support

Single cell sequencing technology can detect the advantages of a small number of cells, which can be applied to prenatal diagnosis and assisted reproduction. Detection of female egg cell polar cells or embryonic cells by single-cell sequencing to select healthy embryo transfer can reduce the birth rate of newborns with congenital genetic diseases and help prevent genetic diseases [46, 47].

### Embryonic cell

In terms of animals, single-cell transcriptome sequencing technology is used to map the cell development of zebrafish and African cockroach embryos, providing important clues for understanding developmental biology [48–50]. Li et al. [51] mapped the genome-wide map of human embryos prior to implantation by single-cell multi-sequence sequencing. This study has implications for the analysis of complex and highly coordinated epigenetic processes in the pre-implantation development of human embryos. Vento-Tormo et al. [52] performed a transcriptome analysis of placental cells in early pregnancy by single-cell sequencing technology and mapped placental cell maps. Through the cell map, three subpopulations of perivascular and stromal cells located in different decidual layers and dNK (decidual natural killer) were found. It has also identified regulatory responses that may minimize the immune response of harmful mothers, as well as revealing interactions that contribute to the success of placenta and reproduction. The findings are important for understanding early pregnancy processes and improving the diagnosis and treatment of pregnancy-related diseases.

## Applications in the field of immunology

As an important system for the body to perform immune response and immune function, the immune system has an important role in resisting the invasion of external pathogens. Many immune cells with unique functions in the immune system are a research hotspot.

### Application of natural killer (NK) cells, DC cells and lymphocytes

Single-cell sequencing technologies can detect individual immune cells, thereby distinguishing different groups of immune cells, as well as discovering new immune cell populations and their relationships (Fig. 2). This helps to understand the complex immune system and propose new targets for disease treatment. Crinier et al. [53] identified subpopulations of the spleen and blood NK cells in mice and humans by single-cell RNA sequencing, revealing two distinct features that distinguish blood and spleen NK cells. And through the comparison of transcriptomics, the similarity between two major subgroups NK1 and NK2 in organs and species is highlighted. This study provides an in-depth understanding of the biology of NK cells and contributes to the translation of animal studies into human-related research. Villani et al. [54] identified multiple subtypes of DC cells and monocytes in human blood by single-cell RNA-seq and revealed a new subset of DC cells that have the properties of plasmacytoid DCs but are effective in activating T cells. In response to this finding, one of the researchers suggested “stimulating such cells or potentially enhancing the body’s immune system to fight cancer.” Such cells may become a new anti-cancer means to remove tumor cells through their own immune system, avoiding the damage of normal chemotherapy drugs to normal cells. The study redefines the relationship among DC cells, helps analyze the developmental processes and functions of the immune system, and completes immune surveillance under normal and disease states. Xin et al. [55] used single-cell RNA sequencing to study immune cells and cytokines during persistent infection. It was found that the heterogeneity of IL-10 expressing CD4 T cells and the production of IL-10 by a subset of helper cells during different infections play an important role in promoting humoral immunity.

### Causes of immune cell heterogeneity

Single-cell sequencing can study immune cells with high heterogeneity caused by pathogens, accurately detect the genetic material of individual immune cells, and help to understand the complex immune mechanism of the body [56]. Interestingly, in addition to pathogens, aging can also lead to increased cellular heterogeneity. Martinez-Jimenez et al. [57] performed single-cell RNA sequencing on CD4+ T cells in different states of young and old mice, and found that aging affects cell transcriptional kinetics, resulting in increased heterogeneity of gene expression among immune cells. Further, the immune cells are unable to make an asynchronous reaction, and the immune performance is weakened. This study helps explain the weakening of the immune system with increasing age. It is suggested that the increase in intercellular transcriptional variation may represent the aging characteristics, laying the foundation for exploring the aging mechanism of cells. Recently, single-cell sequencing has found new discoveries in the immune cells of the central nervous system during the inflammatory phase. Jordão et al. [58] found that in the development of neuroinflammatory pathology, the specific differentiation of myeloid cell subpopulations is obvious because the self-renewal, random proliferation and clonal expansion of CNS macrophage subpopulations. Moreover, the antigen presentation of CNS macrophages has no significant relationship with the pathological role of neuroinflammation. Studies have shown that dendritic cells and monocyte-derived cells are the main participants in antigen presentation during experimental autoimmune encephalomyelitis (EAE). This study maps the dynamic map of myeloid cell subpopulations in the central nervous system, reveals complex changes in the characteristic molecules of myeloid cell subsets during neuroinflammation, and provides a theoretical basis for improving the treatment of EAE.

According to the above research, the heterogeneity of immune cells will affect the immune status of the body. An increase in the heterogeneity of immune cells may cause immune cells to have specific functions that help fight against pathogen invasion. It may also cause the body’s immunity to decline, which is a feature of tissue aging. Therefore, the study of immune cell heterogeneity can help people understand the complex and delicate immune mechanisms of the body, and perhaps adjust some immune mechanisms to treat certain diseases.

## Application in the digestive system and urinary system

Haber et al. [59] found many new intestinal epithelial cell subtypes by single-cell transcriptome sequencing and mapped the expression of intestinal epithelial cells. From this map, the mechanism of intestinal cells maintaining homeostasis and responding to pathogenic microorganisms is explained. Gao et al. [60] used high-precision single-cell transcriptome sequencing to analyze the four digestive organs of human embryonic stage and multiple cells of the adult large intestine, revealing the related mechanisms of gene regulation in the development of four human digestive organs (Fig. 2). Wang et al. [61] demonstrated the heterogeneity of precursor cells forming the initial nephron in the human embryonic stage and the corresponding transcriptional regulatory events and signaling pathways in the process of differentiation of precursor cells into tubular epithelial cells by single-cell transcriptome sequencing (Fig. 2). This study also demonstrated the expression characteristics of candidate pathogenic genes for congenital nephropathy and contributed to the treatment of congenital nephropathy.

## Conclusion and perspectives

After completing the Human Genome Project, scientists have proposed a human cell atlas program designed to complete the mapping of 37 trillion cells in the human body. Single-cell sequencing technologies can accurately study individual cells and will be an important driving force for this project. The cell maps depicted by single-cell sequencing technology help people distinguish among cell types and understand cell-to-cell relationships. One can further understand physiological processes and pathological mechanisms at the single-cell level to find new diagnostic markers or new therapeutic targets. This will provide a practical basis for improving the diagnosis and treatment of the diseases.

This review introduced single-cell sequencing methods and their applications in tumors, microorganism, nervous system, reproductive medicine and immunology, highlighting the great advantages of single-cell sequencing technologies in the study of highly heterogeneous single-cell. However, single-cell sequencing technologies still have problems such as cumbersome operation and high detection cost, which limits the promotion of technology. It is hoped that the single-cell sequencing technologies will be more simplified, more powerful, and the detection cost will be further reduced, so that the technologies can be applied to basic research and play an important role in clinical diagnosis and treatment.

## Data Availability

Not applicable.

## Abbreviations

SCI-seq: single-cell combinatorial marker sequencing technique
scCOOL-seq: single-cell chromatin overall omic-scale landscape sequencing
TSCS: topographic Single Cell Sequencing
scRNA-seq: single-cell RNA-seq
CRISPR: clustered regularly interspaced short palindromic repeats
CROP-seq: cRISPR droplet sequencing
sNuc-Seq: single-Nucleus RNA Sequencing
snDrop-seq: single-core sequencing
scTHS-seq: single-cell transposon hypersensitive site sequencing
TCR: T cell receptor
ERα: estrogen receptor alpha
PPAT: phosphoribosyl pyrophosphate amidotransferase
AZIN1: antizyme inhibitor 1
IDH: isocitrate dehydrogenase
HNSCC: head and neck squamous cell carcinoma
AML: acute myeloid leukemia
ET: essential thrombocythemia
T-ALL: T cell acute lymphoblastic leukemia
NOA: non obstructive azoospermic
dNK cells: decidual natural killer cells
NK cells: natural killer cell
DC cells: dendritic cells
IL-10: interleukin-10
CD4: cluster of differentiation 4
CNS: central nervous system
EAE: experimental autoimmune encephalomyelitis
